# Anticonvulsant effect of liraglutide, GLP-1 agonist by averting a change in GABA and brain glutathione level on picrotoxin-induced seizures

**DOI:** 10.17179/excli2017-283

**Published:** 2017-05-19

**Authors:** Gaurav Gupta, Rajiv Dahiya, Kamal Dua, Dinesh Kumar Chellappan, Juhi Tiwari, Ganesh Narayan Sharma, Santosh Kumar Singh, Anurag Mishra, Rakesh Kumar Sharma, Mohit Agrawal

**Affiliations:** 1 School of Pharmacy, Jaipur National University, Jagatpura 302017, Jaipur, India; 2 School of Medicine and Public Health, University of Newcastle, Newcastle, NSW 2308, Australia; 3 Laboratory of Peptide Research and Development, School of Pharmacy, Faculty of Medical Sciences, The University of the West Indies, St. Augustine, Trinidad & Tobago, West Indies; 4 Discipline of Pharmacy, Graduate School of Health, University of Technology Sydney, Sydney, NSW 2007, Australia; 5 Department of Life Science, International Medical University, Bukit Jalil, Kuala Lumpur, Malaysia; 6 School of Pharmacy, Suresh Gyan Vihar University, Jagatpura 302017, Jaipur, India

## ⁯

Dear Editor,

Epilepsy is the third main accountable cause for neurological debility around the world and is showed via reappearance of unprovoked seizures and resultant distressing consequences in patients and their caretakers. Around seventy million individuals all over the globe have epilepsy, and ninety percent of them are placed in developing international locations (Amudhan et al., 2015[1]). It is well known that liraglutide is primarily employed in the management of type 2 diabetes mellitus. The drug acts as a GLP-1 agonist whereby it exerts its principal mechanism of action. The drug works by increasing β cell targeted release of insulin. The eventual synergistic effects result in an enhanced glycemic management combined with better lipid digestion (Ladenheim, 2015[4]). However, it was recently discovered that, in addition to its antidiabetic potential, it also has a potent neuroprotective function. Much of this is achieved by the ability of the drug to enhance the retention capability of memory in mice. Moreover, studies done on mice have confirmed the effectiveness of liraglutide in improving the total hippocampal pyramidal neuronal number (Hansen et al., 2015[3]). The goal of this research was therefore to study the anticonvulsant potential of this drug on picrotoxin-induced seizures in mice. The study was conducted in albino mice that weighed between 20 g and 25 g. The animals were housed carefully in standard mice cages with the usual mice bedding made of husk. A controlled temperature of 24 ± 2 °C and a relative humidity range of 30-70 % were maintained when the animals were housed in the cages. In addition, a 12 h:12 h light and dark cycle were also maintained. The mice were given free access to drinking water and commercial mice pellet. We used four sets of mice with six mice in each set (n=6). The first set of mice were administered with 0.9 % (w/v) normal saline. These mice served as normal control animals and were administered a dose of 1 mL/100 g of normal saline. The second set of mice were administered with standard clonazepam of 1 mg/kg. This group of animals was labeled as positive control animals. The third and fourth sets of mice were administered with 100 µg/kg and 200 µg/kg of liraglutide respectively. All the drugs were administered through intraperitoneal route. 

The anticonvulsant potential of liraglutide was established through Picrotoxin-induced seizures. Thirty minutes after the drugs were administered, the mice were subjected to the induction of clonic seizures with the help of a single dose of 7.5 mg/kg of picrotoxin. The protective effect of the test drugs was measured through observation of the mice for a period of 15 minutes. The number of mice that failed to produce a convulsion within the 15 minutes of observation time was marked as protected (Taiwe et al., 2016[5]). Time durations of onset and that of clonic or tonic seizures were also measured. After all, observations concluded, the mice were sacrificed by decapitation. The brains from all animals were extracted. The cerebral hemispheres from the extracted brains were separated into two separate parts; left and right brain hemisphere tissues. Reduced glutathione studies were done on one of the parts and GABA studies were performed on the other part. Both studies involved preparation of a 10 % w/v homogenate, formulated using ice cold 0.1 M phosphate buffer (pH 7.4) and 0.01M hydrochloric acid respectively. 

The measurement for brain reduced glutathione (GSH) levels was performed by initially mixing the preformulated brain homogenate with an equal amount of 10 % trichloroacetic acid and then removing the unwanted proteins through centrifugation and then collecting the supernatant mixture which is devoid of any proteins. The supernatant obtained (100 µl) is then mixed with 2 mL of 0.3 M phosphate buffer (pH 8.4), 0.5 ml of 0.04 % DTNB in 1 % trisodium citrate and 0.4 ml of double distilled water. These were added in succession. To measure the amount of GSH in the samples (expressed in µg/g of wet tissue), absorbances at 412 nm were measured on a spectrophotometer. A standard GSH absorbance measurement was also performed. All absorbances were measured within 15 minutes of preparation of the samples (Ellman et al., 1961[2]). On the other hand, for GABA measurement, the brain homogenate was mixed with 16 mL of ice-cold absolute alcohol inside a bottle, which was then centrifuged for 10 minutes at 10000 g. The resultant precipitate was collected and washed 3 times with 10 mL of 90 % alcohol. The washed liquids were pooled along with the supernatant. This mixture was evaporated to dryness on a Petri plate at 70 °C. After this, 2 mL water and 4 mL of chloroform were added to the dry substance, which was centrifuged at 182 g. The supernatant containing GABA was then collected. This was then loaded on a Whatman paper (No. 41) and was subjected to chromatography. The mobile phase constituted of n-butanol: acetic acid: water in the ratio of 50 mL:12 mL:60 mL. Paper chromatography was performed using the ascending technique. The spraying agent used was 5 % ninhydrin solution in 95 % ethanol. The chromatograms were then dried for 1 h at 90 °C. The blue color spot which developed was cut and heated with 2 mL ninhydrin solution on a water bath for 5 min. A small quantity of water (10.0 mL) was added to the solution which was kept for 1 h. Eventually, 4 mL of the supernatant was decanted for which the absorbance was measured at 570 nm (Tamboli et al., 2012[6]).

Picrotoxin (7.5 mg/kg, i.p.) resulted in tonic convulsions followed by 100 % mortality. Treatment with clonazepam (1 mg/kg) and liraglutide (100 and 200 mg/kg, i.p.) per se significantly delayed onset of convulsions (p < 0.05, p < 0.01 and p < 0.001, respectively). The test doses also significantly reduced (p < 0.05 and p < 0.001, respectively) the duration of tonic convulsions and reduced mortality in mice as compared to picrotoxin-treated control mice. Administration of clonazepam (1 mg/kg) and liraglutide (100 and 200 mg/kg, i.p.) per se significantly increased GABA levels (p < 0.01 and p < 0.001, respectively) and GSH levels (p < 0.01 and p < 0.001 respectively) compared to picrotoxin-treated control (Table 1(Tab. 1)). It was concluded that liraglutide showed significant antiepileptic activity in mice probably through an increase in brain GABA and GSH levels. Further studies are necessary to research the details and the mechanism of action of this drug so that this drug could be used effectively in the management of epilepsy in human beings. 

## Conflict of interest

The authors declare no conflict of interest.

## Figures and Tables

**Table 1:**
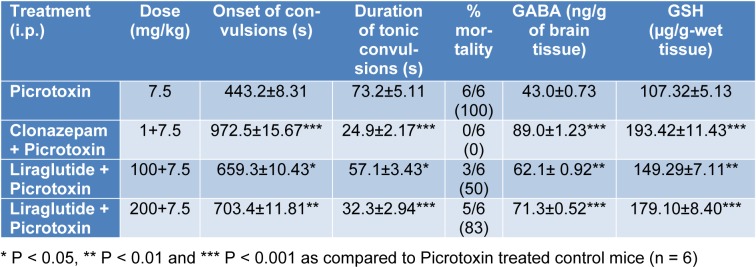
Effect of liraglutide and clonazepam on picrotoxin-induced convulsions, the level of Brain GABA and GSH in mice
